# Selected Parameters of Bone Turnover in Neuroendocrine Tumors—A Potential Clinical Use?

**DOI:** 10.3390/jcm12144608

**Published:** 2023-07-11

**Authors:** Janusz Strzelczyk, Monika Wójcik-Giertuga, Joanna Katarzyna Strzelczyk, Alicja Prawdzic Seńkowska, Krzysztof Biernacki, Beata Kos-Kudła

**Affiliations:** 1 Department of Endocrinology and Neuroendocrine Tumors, Department of Pathophysiology and Endocrinology, Faculty of Medical Sciences in Zabrze, Medical University of Silesia in Katowice, 35 Ceglana St., 40-514 Katowice, Poland; 2 Department of Medical and Molecular Biology, Faculty of Medical Sciences in Zabrze, Medical University of Silesia, 19 Jordana St., 41-808 Zabrze, Poland

**Keywords:** bone turnover markers, neuroendocrine tumors (NET), osteocalcin (OST), osteoprotegerin (OPG), insulin-like growth factor binding protein 3 (IGFBP-3)

## Abstract

Background: Currently, there are no effective markers to diagnose and monitor patients with neuroendocrine tumors (NETs). The aim of this study was to assess bone metabolism based on selected markers of bone turnover: OST, OPG, and IGFBP-3, in both the group of patients with NETs and the control group. Associations with selected sociodemographic, biochemical, and clinicopathological characteristics were examined. We also evaluated any potential associations between these markers and selected biochemical markers of NETs commonly used in clinical practice. Methods: The study group included 60 patients with GEP-NETs and BP-NETs, while the control group comprised 62 healthy individuals. The serum concentrations of OST, OPG and IGFBP-3 were assessed using ELISA. Results: OST and OPG levels were significantly higher in the study group compared to the control group. In the study group, we observed a significant correlation between OPG and the clinical stage and chromogranin A. Additionally, an association was found between OPG and histological grade, Ki-67, and metastasis in GEP-NET cases. Conclusions: Markers of bone turnover cannot be used in the routine diagnostics of neuroendocrine tumors. Nonetheless, these markers may help evaluate the skeletal system in patients with NETs. Further research is needed to determine the utility of osteocalcin (OST) and osteoprotegerin (OPG) as potential biomarkers for neuroendocrine tumors.

## 1. Introduction

Neuroendocrine tumors (NETs) are a heterogeneous group of tumors that originate from neuroendocrine cells. They are typically solid tumors and account for approximately 2% of all malignant tumors. The incidence of these cancers is constantly increasing [1]. The vast majority of them are located in the gastrointestinal tract (gastro-entero-pancreatic neuroendocrine tumor—GEP-NET), which make up for 70% of all NETs. Less commonly, they are localized in the respiratory system (BP-NET—bronchopulmonary neuroendocrine tumors) and occasionally in the urogenital system [1,2]. These tumors are usually slow-growing and have a more favorable prognosis compared to other malignant tumors. Biochemical diagnostics in NETs involve the use of non-specific markers, such as serum chromogranin A concentration, which is frequently used for diagnosis and patient monitoring [1]. Currently, there are no effective markers for diagnosing and monitoring neuroendocrine tumors, including early detection of progression. Selecting a specific marker for an NET requires considering the suspected tumor type’s location. The most common symptom complex associated with these tumors is carcinoid syndrome, which in its classic form is mainly dependent on excessive serotonin secretion. The gold standard for screening for carcinoid syndrome is the evaluation of serotonin metabolite levels, i.e., the daily collection of urine for 5-hydroxyindole acetic acid (5-HIAA) [1,3]. 

Many factors influence the continuous processes of bone formation and bone resorption. The course of these states can be assessed using biochemical markers of bone turnover. We distinguish markers of bone formation and bone resection. The markers of bone formation include osteocalcin (OST), N-terminal propeptide of procollagen I (PINP), and bone fraction of alkaline phosphatase (b-ALP). Resorption markers include C-terminal cross-linked telopeptide of type I collagen alpha chain (CTx), N-terminal cross-linked telopeptide of type I alpha chain (NTx), pyridinoline (PYD), and deoxypyridoline (DPD) [4,5]. Osteoblasts are responsible for the synthesis of the osteoid, which is made of compounds containing proteoglycans and collagen. Osteocalcin, as mentioned above, is a marker of bone formation and belongs to the proteins of the bone matrix. It is synthesized by not only osteoblasts, but also chondrocytes and odontoblasts. It is responsible for calcium binding and matrix mineralization [5]. Osteoclasts account for bone resorption, which is necessary to maintain the proper mechanical function of bone. They release hydrolases, which in turn dissolve the components of the extracellular matrix. Osteoclasts are regulated by osteoblasts via the receptor activator of nuclear factor κ-B ligand (RANKL)-receptor activator of nuclear factor κ-B (RANK) signaling pathway [6]. Osteoprotegerin (OPG) is a protein produced by osteoblasts which plays an important role in bone turnover by inhibiting osteoclastogenesis and thus bone resorption. It serves as a decoy receptor which binds and opposes RANKL. Therefore, the ratio of RANKL to the concentration of OPG determines and greatly influences the differentiation of osteoclasts [7]. The key hormones that influence bone metabolism are parathyroid hormone, calcitonin, and vitamin D3. Glucocorticosteroids have a catabolic effect on bone by stimulating osteoclasts and inhibiting the expression of the OPG gene, thereby increasing bone resorption [8]. Hormones with an anabolic effect on the bone include sex hormones, insulin, and growth hormone. Growth hormone and insulin-like growth factor 1 (IGF-1) have a major impact on the metabolism of bone tissue and are responsible for promoting osteoblast differentiation and thus accelerating bone growth. The concentration of insulin-like growth factor binding protein 3 (IGFBP-3), which plays a crucial role in binding and regulating the activity of IGF-1, was determined in this study. According to a recent study, IGFBP-3, apart from stabilizing and transporting IGF-1, influences the differentiation of osteoblasts [9]. Bone turnover markers, produced by osteoblasts and osteoclasts, play an unquestionable role in the management of osteoporosis [10]. These markers can also be used in imaging of the bone remodeling process and aid in the diagnosis of cancer patients. However, diagnostic markers specific to NETs are still being investigated. Patients diagnosed with GEP-NETs have an increased risk of developing osteopenia and osteoporosis [11,12]. The aim of this study was to assess bone metabolism using selected markers of bone turnover: OST, OPG, and IGFBP-3, in patients with NETs and in the control group. Additionally, we aimed to evaluate the association of these markers with selected sociodemographic, biochemical, and clinicopathological characteristics.

## 2. Methods

All patients were recruited at the Department of Endocrinology and Neuroendocrine Tumors, Medical University of Silesia in Katowice (Poland). The study was conducted in accordance with the Declaration of Helsinki and good clinical practice guidelines. The Ethics Committee of the Medical University of Silesia (No. PCN/CBN/0052/KB1/24/II/22 and KNW/0022/KB1/137/I/14) approved this study. The main inclusion criteria for the study group were a diagnosis of neuroendocrine tumor, age above 18, absence of chronic disease requiring pharmacotherapy, and signed informed consent to participate in the study. Study group included patients before the initiation of treatment. Exclusion criteria for both groups were pregnancy, lactation, age less than 18, and lack of informed consent to participate in the study. From all participants, peripheral blood was collected into S-Monovette tubes with a clotting activator (Sarstedt, Germany). To obtain serum, the stored blood was centrifuged at 4 °C and 1500 rpm for 15 min, and then the serum was stored at −80 °C for further analysis. The serum concentrations of osteocalcin (OST), osteoprotegerin (OPG), and insulin-like growth factor binding protein 3 (IGFBP-3) were assessed using the ELISA method.

### 2.1. Study Group

Study group included 60 patients (38 women and 22 men) with neuroendocrine tumors of the digestive tract (GEP-NETs) and the respiratory system (BP-NETs) with a mean age of 58.02 ± 12.9 years and mean BMI of 25.34 ± 4.96. Clinical information about the patients was retrieved from their hospital records. Information on the location of the primary lesion, histological grade of the neoplasm, clinical stage, Ki-67 proliferation index, and metastasis is presented in Table 1. Detailed information about demographic data and measured biochemical parameters is shown in Table 2.

### 2.2. Control Group

Control group comprised 62 healthy individuals (46 women and 16 men) with a mean age of 35.97 ± 11.5 years and mean BMI of 29.29 ± 11.47. Detailed information about demographic data and measured biochemic parameters is presented in Table 2. Chromogranin A, serotonin, and 5-HIAA levels were not measured in the control group.

### 2.3. Enzyme-Linked Immunosorbent Assay (ELISA)

The serum concentrations of osteocalcin (OST), osteoprotegerin (OPG), and insulin-like growth factor binding protein 3 (IGFBP-3) were assessed using the ELISA method, adapting the procedures described by the producers. The following tests were used: for OST, it was BioSource hOST- EASIA Kit, BioSource Europe S.A., Nivelles, Belgium (catalog number KAP1381); for OPG, Human Osteoprotegerin Elisa BioVendor, Modrice, Czech Republic (catalog number RD194003200); and IGFBP-3 Human IGFBP-3 Immunoassay the R&D Systems, Abingdon, UK (catalog number DGB300). ELISA tests plates were read by Bio-Tek µQuant Universal Microplate Spectrophotometer (Bio-Tek, Winooski, VT, USA), using 450 nm as the primary wavelength. Data Analysis Software KCJunior v. 1.41 (Bio-Tek, USA) was used. The absorbance was transformed to a concentration of pmol/L for OPG and ng/mL for OST and IGFBP-3. All standards and serum samples were run in duplicates.

### 2.4. Statistical Analysis

Comparisons between study groups and control group for patients’ characteristics were performed using the Kruskal–Wallis test and the Spearman rank correlation coefficient (r_S_) was calculated for them. All values are presented as mean ± standard deviation. Statistical analysis was performed using Microsoft Excel v. 2108 (14332.20503) (Microsoft, Redmond, WA, USA) and Statistica v. 13.36.0 (StatSoft, Kraków, Poland). ROC analysis for osteocalcin, osteoprotegerin, and insulin-like growth factor binding protein 3 was performed with easyROC curve analysis web-tool version 1.3.1 [12] using default settings. In all tests, a value of *p* < 0.05 was considered statistically significant.

## 3. Results

Levels of osteocalcin and osteoprotegrin were significantly higher in the study group compared with the control group (*p* = 0.001 and *p* = 0.003, respectively) while the level of IGFBP-3 did not differ between the two groups (Table 3). 

The age of the study group compared with the control was significantly higher. However, there were no significant differences in BMI, glucose, total cholesterol, or triglycerides levels between study group and control group (Table 2). 

In the study group, we observed a positive correlation between osteocalcin and calcium levels (r_S_ = 0.28, *p* = 0.031), but no correlation between osteocalcin and sex, age, BMI, levels of chromogranin A, serotonin, 5-hydroxyindole acetic acid, phosphate, cortisol, glucose, total cholesterol, triglycerides, osteoprotegerin, or IGFBP-3. Moreover, there were no significant associations between the levels of osteocalcin and the location of the primary lesion, histological grade, clinical stage, Ki-67, and metastasis in the study group. 

In the control group, there were no correlations between osteocalcin levels and sex, and age. We observed a significant positive correlation between osteocalcin and IGFBP-3 (r_S_ = 0.48, *p* = 0.002). Additionally, we found a positive correlation between osteocalcin and BMI, although it did not reach statistical significance (r_S_ = 0.56, *p* = 0.089). Furthermore, there was a negative correlation between osteocalcin and glucose levels, which also did not reach statistical significance (r_S_ = −0.6, *p* = 0.067). We did not find any correlations between osteocalcin and the level of total cholesterol or triglycerides.

In the study group, osteoprotegerin positively correlated with age (r_S_ = 0.48, *p* = 0.001), but no correlation was observed between osteoprotegerin and sex, or BMI and the levels of glucose, total cholesterol, or triglycerides. There was no significant association between levels of osteoprotegerin and the location of the primary lesion. In the study group, we observed a significant correlation between OPG and clinical stage (r_S_ = 0.47, *p* < 0.001), and chromogranin A (r_S_ = 0.42, *p* = 0.001), but no significant correlation with serotonin, 5-HIAA, calcium, phosphate, or cortisol levels. In GEP-NET patients, NET G1 had lower osteoprotegerin levels than NET G2 and NET G3 patients (NET G1: *n* = 30, mean OPG level: 5.7 ± 3.1, NET G2 and G3: *n* = 12, mean OPG level: 6.4 ± 1.6, *p* = 0.050), whereas NET G2 had higher osteoprotegerin levels than NET G1 and NET G3 (NET G2: *n* = 7, mean OPG level: 6.9 ± 1.3, NET G1 and G3: *n* = 35, mean OPG level: 5.7 ± 2.9, *p* = 0.014). There was no significant correlation between OPG levels and histological grade for BP-NET cases. Patients in clinical stage IV had higher levels of OPG (clinical stage IV: *n* = 29, mean OPG level: 6.5 ± 3.0, clinical stage I-III: *n* = 31, mean OPG level: 4.9 ± 1.8, *p* = 0.001), whereas in clinical stage I lower levels of OPG were observed (clinical stage I: *n* = 28, mean OPG level: 4.8 ± 1.9, clinical stage II-IV: *n* = 32, mean OPG level: 6.5 ± 2.8, *p* < 0.001). Patients with Ki-67 below 3% had significantly lower levels of osteoprotegerin (Ki-67 < 3%: *n* = 18, mean OPG level: 4.7 ± 1.7, other participants *n* = 42, mean OPG level: 6.1 ± 2.8, *p* = 0.011), but patients with Ki-67 between 3% and 20% had significantly higher levels of osteoprotegerin (Ki-67 = 3% to 20%: *n* = 6, mean OPG level: 7.0 ± 1.4, other participants *n* = 54, mean OPG level: 5.6 ± 2.6, *p* = 0.026). Metastatic patients (*n* = 30, mean OPG level: 6.6 ± 2.9) had a higher level of osteoprotegerin than patients with no metastasis (*n* = 30, mean OPG level: 4.8 ± 1.8, *p* = 0.001).

In the control group, there was no association between osteoprotegerin and sex, age, BMI, and total cholesterol. However, OPG showed a positive correlation with glucose levels (r_S_ = 0.7, *p* = 0.024), and there was a positive correlation with triglycerides levels, although not statistically significant (r_S_ = 0.6, *p* = 0.067). 

Levels of IGFBP-3 in the study group negatively corelated with age (r_S_ = −0.35, *p* = 0.031) and positively with triglycerides (r_S_ = 0.41, *p* = 0.012), but there were no significant correlations with sex, BMI, glucose, and total cholesterol levels and chromogranin A, serotonin, 5-hydroxyindole acetic acid, calcium, phosphate, or cortisol levels in the study group. There were no significant associations between levels of IGFBP-3 and the location of the primary lesion, histological grade, clinical stage, Ki-67, and metastasis in the study group.

In the control group, IGFBP-3 negatively corelated with BMI (r_S_ = −0.41, *p* = 0.02) but no correlation was observed between IGFBP-3 and sex, age, the levels of glucose, total cholesterol, and triglycerides.

There were no correlations between osteocalcin, osteoprotegerin, or IGFBP-3 in the study and control group. There were no significant differences in the levels of osteocalcin, or osteoprotegerin and IGFBP-3 between those with and without bone metastases.

Spearman rank correlation coefficients r_S_ and *p* values for study group and control group are attached as Supplementary Materials (Supplementary Table S1a for study group and Table S1b for control group).

**Figure 1:**
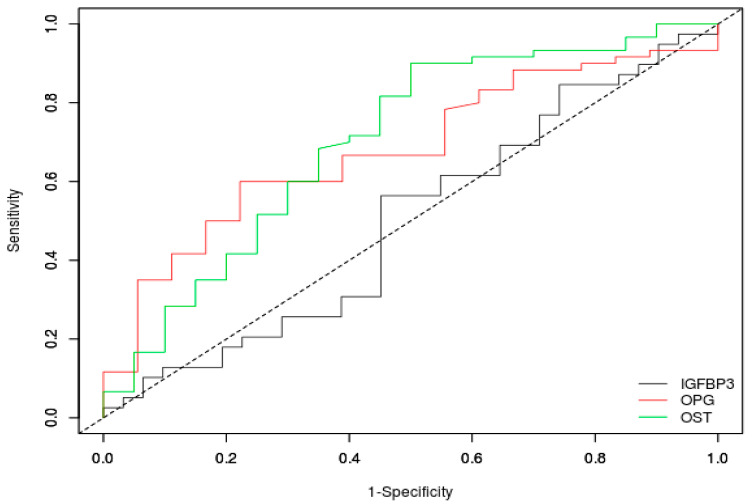
ROC curves for osteocalcin (OST), osteoprotegerin (OPG), and insulin-like growth factor binding protein 3 (IGFBP-3) in the study and control groups.

**Figure 2:**
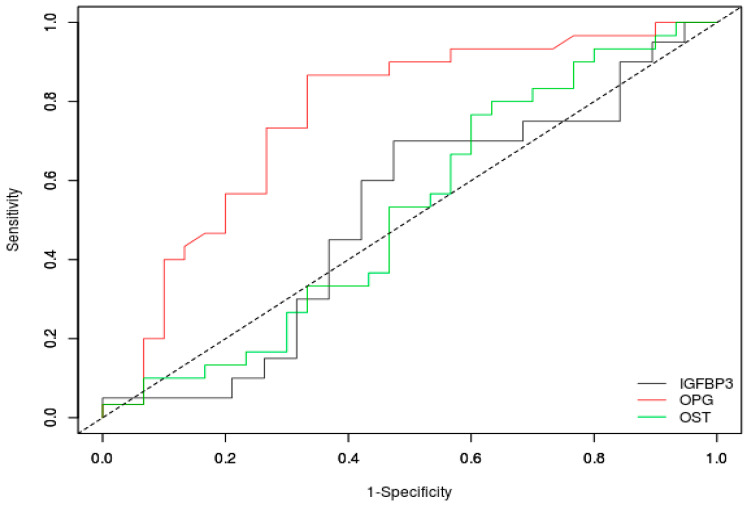
ROC curves for osteocalcin (OST), osteoprotegerin (OPG), and insulin-like growth factor binding protein 3 (IGFBP-3) in the study groups with metastasis and without metastasis.

ROC analysis of the diagnostic potential of osteocalcin, osteoprotegerin, and IGFBP-3 showed that osteocalcin (AUC = 0.701, cut-off value: 5.88, *p*-value < 0.001) and osteoprotegerin (AUC = 0.681, cut-off value: 4.76, *p*-value = 0.001) may have a diagnostic potential to identify NET patients, while IGFBP-3 does not (AUC = 0.498, cut-off value: 2754.8, *p*-value = 0.972). ROC curve plots are presented in Figure 1. Roc analysis also showed that osteoprotegerin may have some diagnostic potential in terms of detecting the presence of metastasis (AUC = 0.761, cut-off value: 4.76, *p*-value < 0.001), while osteocalcin (AUC = 0.520, cut-off value: 7.96, *p*-value = 0.795) and IGFBP-3 (AUC = 0.511, cut-off value: 2601.7, *p*-value = 0.914) do not. ROC curve plots are presented in Figure 2.

## 4. Discussion

Bone turnover markers, which are products of osteoblasts and osteoclasts, are an essential role in the management of osteoporosis [10]. However, the continuous processes of bone formation and bone resorption can also be disturbed in cancer patients. Patients diagnosed with GEP-NETs have an increased risk of developing osteopenia and osteoporosis [11,12]. Hormones secreted by functioning GEP-NETs that may affect bone metabolism are serotonin, cortisol, and parathyroid hormone-related peptide. Additionally, patients suffering from GEP-NETs are at higher risk of malnutrition and consequent development of vitamin D deficiency [11]. Several studies have aimed to understand bone metabolism in NETs and identify specific markers, although the exact mechanism is still unknown. Neuroendocrine tumors can be either hormonally functioning or non-functioning. The proposed mechanisms explaining osteoporosis in NENs (neuroendocrine neoplasms) include excessive secretion of cortisol (ectopic Cuhing’s syndrome) and carcinoid syndrome associated with excess of serotonin secreted by NENs [11,12]. Other factors related to osteoporosis and the increased risk of fractures in patients with NENs include advanced age, malabsorption syndrome related to surgical treatment or treatment with somatostatin analogues, and the frequent presence of bone metastases [11,12]. Currently, there are no specific recommendations for assessing bone turnover in these patients, but such evaluations might improve the prevention of severe complications like bone fractures, ultimately enhancing the quality of life [11].

Osteocalcin is a non-collagen protein that is responsible not only for bone mineralization but also for systemic glucose and energy metabolism [13,14]. According to Diemar et al., OST concentrations decrease with age and are significantly higher in females [15]. However, in our study, we did not observe a correlation between OST and age and sex. Disturbances in bone turnover are observed in severe obesity [16]. OST can affect energy metabolism in various ways, with its main role being associated with osteoblast function, which can be regulated by leptin through various central hypothalamic pathways [17]. Moon et al. suggested that decreased OST levels were independent of menopausal status in women and were significantly associated with metabolic syndrome [14]. Diemar et al. reported decreased OST levels in patients with increased BMI [15], while Perfetto et al. demonstrated no association between obesity status and osteocalcin levels in postmenopausal women [18]. In our study, we observed a positive correlation between osteocalcin and BMI, and a negative correlation between OST and glucose levels in the control group, although both correlations did not reach statistical significance. In diabetes, low bone turnover is observed, leading to decreased serum levels of bone formation markers and bone resorption markers, such as OC and CTX [19]. Moreover, OST may play a role in lipid metabolism and may reduce lipogenesis in the liver [20]. However, we did not confirm the correlation between OST and lipid profile in our study.

Osteocalcin has been the subject of interest in various research studies, including cancer [21,22,23]. In a study on advanced prostate cancer, serum OST levels were correlated with bone metastases and disease recurrence after hormonal treatment. Among 27 patients with bone metastases, 22 showed increased OST levels [24]. Contrarily, lower concentrations of OST were detected in the serum of patients with non-small cell lung cancer with bone metastases [25,26]. However, other studies in lung cancer did not find differences in the concentrations of OST and β-CTx (β-form of C terminal telopeptide) between patients with bone metastases and patients without bone metastases [26,27]. In a separate study, researchers investigated diagnostic value of circulating osteocalcin-positive cells (cOC) in breast cancer with bone metastasis. They discovered that cOC may be useful as a biomarker for early diagnosis and progression of bone metastasis [28]. Delaka et al. observed an association between levels of serum OC and CTx and bisphosphonate-related osteonecrosis of the jaw in high-risk prostate cancer patients [29]. On the contrary, Bager et al. revealed that decreased levels of osteocalcin and CTX-1 were associated with an increased risk of all types of cancer in postmenopausal women [30]. Van Dijk et al. assessed the effect of secretion of the serotonin metabolite 5-hydroxyindoleacetic acid (5-HIAA) on bone metabolism in carcinoid syndrome [31]. They determined the concentration of 5-HIAA in the urine of patients with and without carcinoid syndrome. They also assessed selected markers of bone formation (including OST) and bone resorption. However, no significant differences were found between the group of “secreting” and the control group of “non-secreting” [31]. The researchers suggested that receptors for serotonin are present in bone cells which may have a significant influence on bone tissue [31]. Nonetheless, the duration of the elevated serotonin levels was not specified in their study, and it may be too short to have an effect on bone metabolism. Walsh et al. assessed the levels of serotonin in the blood and of 5-HIAA in plasma and urine, as well as bone density and bone turnover markers, OST, PINP, and C-terminal cross-linked telopeptide of type I collagen alpha chain (CTX) in patients with carcinoid syndrome and in a group of healthy people [32]. This study showed a higher concentration of OST in the group with carcinoid syndrome compared to the control group. They also found a correlation between the levels of 5-HIAA and OST [32]. Given that OST is a marker of bone formation, the results suggest that bone formation is not inhibited in carcinoid syndrome. The authors concluded that prolonged exposure to serotonin excess did not lead to a significant reduction in bone density. It is hypothesized that there may be other cancer-related factors that protect the skeleton from the damaging effects of high serotonin levels [32]. In our study, we also observed an increased concentration of OST in patients with NETs, but no correlation between OST and chromogranin A, or serotonin and 5-HIAA levels. Moreover, as reported by Walsh et al., in the control group, a correlation was found between the levels of serotonin and OST and other markers of bone turnover, such as PINP and CTX [32]. Considering the above-mentioned presence of serotonin receptors on osteoblasts, it is possible that OST influences these cells. Nevertheless, despite the long exposure to high concentrations of circulating serotonin in the group of patients with carcinoid syndrome, no significant differences were found in bone density and bone structure compared with the control group [32]. However, in acromegaly, in which treatment with a long-acting somatostatin analog (octreotide) is used, a reduction in OST via lowered IGF-1 levels was observed [33]. In our study, we observed some diagnostic potential of OST in patients with NET, as its level was significantly higher in the study group compared with the control. This observation is important and indicates the possibility of using this parameter in everyday clinical practice. As observed in previous studies, the was a lack of decrease in bone density and bone structure in NET patients [32]. However, in our study, we did not perform an analysis of bone density. We speculate that the increased levels of OST, a marker of bone formation, may be related to the lack of decrease in bone density and bone structure in NET patients. Further studies are needed to investigate this aspect in neuroendocrine tumors. It is important to note that some neuroendocrine tumors have a tendency to form calcifications, which have been described in up to 30% of lung carcinoids, for example [34]. Tsubochi et al. reported an interesting case of ossification (which is less common than calcification) occurring in a patient with lung carcinoid [34]. The immunohistochemical analysis revealed that the tumor expressed OST and BMP-2 (bone morphogenetic protein-2), which may be responsible for the process of ossification [34]. We hypothesize that the increased OST levels may result in the calcification and ossification process in other NET patients, including those in our study group. However, we did not confirm the correlation between OST and histological grade, clinical stage, Ki-67, and metastasis. Given the conflicting results in available research, the utility of OST as a potential biomarker of metastatic disease remains uncertain. Nonetheless, in our study, we propose OST as a potential biomarker in NETs, but further research is required to assess its utility in clinical practice.

The RANKL/RANK/OPG pathway is essential for maintaining skeletal integrity under physiological conditions. However, osteoclast-mediated bone resorption also contributes to many skeletal pathologies or skeletal-related events (SREs) in patients with pre-existing cancer. Osteoprotegerin (OPG), a member of the TNF (tumor necrosis factor) receptor family, is involved in the regulation of bone turnover. OPG has been investigated as a potential agent in the treatment of osteoporosis and bone cancer as it acts as an inhibitor of osteoclastogenesis [35]. In our study, we did not confirm the correlation between OPG and sex in both groups, but we observed a positive correlation between OPG and age in the study group. Valentini et al. also confirmed the correlation between age and OPG and it was suggested that it might be related to the Frailty Score in the elderly, regardless of the fracture event. They concluded that OPG may be considered as a frailty marker in these patients [36]. Contrarily, Akhtar et al. reported no correlation between OPG and age [37]. The crucial role of OPG in metabolic homeostasis has been extensively studied. According to Cheng et al., leptin can promote osteoblast mineralization and down-regulate RANKL mRNA expression [38]. Holecki et al. found significantly lower OPG levels in obese individuals compared to those with normal weight [39]. However, in our study, we did not observe differences between OPG and BMI. Valentini et al. demonstrated an association between OPG and glucose metabolism [19]. Chronic hyperglycemia and advanced glycation end products (AGEs) are one of the most important factors responsible for endothelial cell dysfunction and vascular complications [19]. OPG levels were higher in diabetic patients with poor glucose control compared to the control group. The authors showed that OPG was directly associated with HbA1c, especially in non-fractured elderly subjects [19]. We also demonstrated a positive correlation between OPG and glucose levels in the control group. AGEs inhibit osteoblast and osteoclast activities and reduce bone turnover in diabetes patients [19]. Moreover, OPG can be secreted by vascular smooth muscle cells and endothelial cells, which may be related to impaired bone quality and more frequent fractures in these cases [19]. The authors observed an association between higher levels of OPG and fractures. It is hypothesized that OPG may be helpful to limit bone resorption in the early stage of fracture [19]. As it is known, increasing OPG levels inhibit osteoclastogenesis through the mediation of the RANK/RANKL/OPG pathway and might play a role in increasing bone mass [16]. On the contrary, according to Qi et al., in diabetic osteoporosis, it was confirmed that treatment with chondroitin sulfate (which can reduce glucose level) in diabetic rats can increase OPG level and decrease RANKL expression, and in this mechanism may improve the bone microstructure and the bone mineral density [40]. Gohar et al. suggested an association between lipids and OPG in a group of patients with depression and schizophrenia. The authors concluded that this association may result in the inflammatory pathway as they observed higher levels of selected cytokines in both patients with obesity and selected psychiatric disorders [41]. In mice, dyslipidemia can result in elevated OPG levels [41]. In our study, we found a correlation between OPG and triglyceride levels in the control group.

Our study made important observations regarding OPG levels in patients with NETs. OPG levels were significantly higher in NET patients, and we found a positive correlation between OPG and chromogranin A, a non-specific marker commonly used for the diagnosis of NET patients. Currently, there are no effective biomarkers for patients with neuroendocrine tumors, including markers for diagnosis and early detection of progression. In our study, we confirmed the potential utility of OPG as a biomarker for monitoring NET patients, but it requires further research. We are still searching for better parameters that may be useful in clinical practice to monitor patients with NETs. The role of OPG has been extensively investigated in cancer patients, such as in breast cancer, prostate cancer, multiple myeloma, and hepatocellular carcinoma [42]. In tumorigenesis, OPG acts as a decoy receptor for tumor necrosis factor-related apoptosis-inducing ligand (TRAIL) and inhibits TRAIL-induced apoptosis, thereby increasing the survival of neoplastic cells. [42]. TRAIL is an endogenous ligand of OPG which might explain the hypothetical role of OPG in cancer progression [42]. Holen et al. suggested that OPG is an essential survival factor in hormone-resistant prostate cancer cells [43]. Furthermore, OPG seems to play a role in vascular biology; thus, it may ultimately influence the tumor angiogenesis process and its progression [44]. In pancreatic cancer, the association between OPG and cancer progression was also confirmed [45]. Many studies confirmed the relationship between OPG and the clinical stage of the disease. According to Chudecka-Głaz et al., higher OPG values were found in advanced ovarian cancers compared to less advanced stages of the disease [46]. In bladder cancer, high serum OPG levels were linked to advanced tumor stages and significantly lower overall survival [47]. In gastric cancer, higher expression of OPG was more common in stages III and IV than in stages I and II [44]. Osteoprotegerin has been linked to a higher risk of metastasis in patients with early breast cancer [48]. In cervical cancer, high immunohistochemical OPG expression was correlated with tumor grade, presence of metastases, high clinical stage, and altogether poor overall survival [49]. Different studies have reported that lower expression of OPG in breast cancer was associated with significantly better overall survival [50]. Moreover, in non-oncological disorders such as in amyloidosis and chronic kidney disease, levels of OPG correlated with the stage of the disease [51,52]. These studies suggest that OPG may be an effective prognostic factor and may indicate generalized inflammation and vascular defect in primary systemic amyloidosis [51]. Younger patients who develop chronic kidney disease may have higher OPG levels due to a worse prognosis. OPG levels were positively associated with the calcium score in these patients [52]. Although this evidence supports the role of OPG in vascular biology, further studies are still needed. In our study, we also observed correlations between OPG and histological grade, clinical stage, Ki-67, and metastasis. In particular, we found some diagnostic potential of OPG in detecting metastasis. Metastatic patients had a higher level of osteoprotegerin than patients with no metastasis. We also noted that NET G2 patients (Ki-67 between 3–20%) had higher OPG levels, whereas NET G1 patients (Ki-67 lower than 3%) had lower OPG levels. The association between OPG and a higher Ki-67 score supports the role of OPG in tumor progression. As it is known, NET G2 patients are characterized by slightly worse prognoses compared to NET G1 patients and therefore require more frequent monitoring to detect progression. We observed higher concentrations of OPG in metastatic disease and thus a diagnostic potential of OPG in the detection of metastatic disease. The impact of neoplastic disease on OPG levels has been extensively described; however, there are conflicting data regarding the association between OPG levels and patients’ survival [53]. For instance, OPG was suggested as a potential biomarker of breast cancer risk [54,55]. Meanwhile, according to Park et al., the inhibition of the RANK pathway in BRCA1 mutation carriers might be used as a preventive treatment for breast cancer since the researchers observed decreased levels of OPG in BRCA1 carriers [54]. Furthermore, OPG has been implicated in the activation of quiescent breast stromal fibroblasts with anticancer activity, suggesting that recombinant OPG protein (rOPG) might be considered a potential treatment of breast cancer [56].

Insulin-like growth factor binding protein (IGFBP-3) plays a role in the differentiation of osteoblasts. It has been shown to influence bone morphogenetic protein (BMP-2), resulting in a reduction in osteoblast differentiation. Furthermore, IGFBP-3 can affect bone formation in an IGF-1-dependent and -independent manner [9]. IGFBP-3 can therefore increase the number of cells in the pre-osteoblastic or immature stage. The IGF-1/IGFBP-3 axis may be responsible for the control of bone metabolism by both inhibiting and accelerating osteoblast differentiation. However, further research is needed to fully understand the role of the IGF-1/IGFBP-3 axis in overall bone metabolism [9]. IGFBP-3 is the most important of the carrier proteins of IGF1 in the blood and plays a crucial role in the metabolism in humans. It has been found to be significantly increased in females [57]. In our study, we did not observe a correlation between IGFBP-3 levels and sex; however, we did find a negative correlation between IGFBP-3 levels and age in the study group. Similarly, Kucera et al. suggested that IGF-1 and IGFBP-3 may decrease with age [57]. Contrarily, Almaki et al. observed that salivary IGFBP-3 levels correlated with age in children and younger adults, and the levels of IGFBP-3 of 13–19-years-old patients were increased compared to the 6–12-years-old patients [58]. This observation may be linked with bone maturity as from prepuberty to the onset of puberty the levels of IGF-1 are elevated and then later in life they decrease [58]. The increased IGFBP-3 concentrations were associated with longer telomeres in older men, independently of age [59]. Telomere shortening is known to contribute to cellular aging, and it is hypothesized that this process is an essential factor in aging-related diseases. The study confirmed additive associations of higher IGF-1 and higher estrogen levels with telomere length [59]. Obese patients have lower IGF-1 during adulthood and obesity has a weaker association with IGFBP-3 compared with IGF-1 [60]. In the literature, there are conflicting results regarding the association between weight status and IGFBP-3, and its influence on metabolic regulation [61]. Therefore, further studies are needed to evaluate this parameter. In our study, we observed that IGFBP-3 negatively correlated with BMI in the control group. Although the role of IGFBP-3 in glucose and lipid metabolism is still not fully understood, it has been suggested that IGFBP-3 may inhibit adipocyte differentiation and may play an important role in the development of insulin resistance [61]. We did not confirm a correlation between IGFBP-3 and glucose levels, but we did demonstrate a positive correlation between IGFBP-3 and triglycerides. Eggert et al. also reported a positive correlation between IGFBP-3 and triglycerides, along with an inverse correlation with HDL levels. The authors observed that IGFBP-3 had a stronger correlation with lipids compared to IGF-1. Contrarily, it has been suggested that that lower HDL levels in elderly result from the decrease in GH and IGFBP-3 levels [62].

Insulin-like growth factor binding protein 3 is known as a p53 tumor suppressor-regulated protein and it acts as a carrier of IGF [63]. IGFBP-3 may play a role in the pathophysiology of prostate, lung, and breast cancer [63]. In the literature, there are conflicting results reporting both suppressing and promoting tumor effects of IGFBP-3. IGFBP-3 may also be involved in anti-metastatic signaling in cancer [63]. Furthermore, an association between IGFBP-3 and cancer risk was confirmed in colorectal cancer [64]. Similarly, IGFBP-3 was significantly increased in the early stages of prostate cancer, providing important evidence about the role of IGF signaling [65]. IGFBP-3 might be also considered a potential diagnostic and prognostic biomarker in the treatment of bone metastases in lung cancer [66]. IGFBP-3 positively correlated with lower clinical stage, Ki-67 staining and grading, and the absence of necrosis and improved overall survival in lung cancer [67]. In a study on Ewing sarcoma, high levels of both circulating IGF-1 and IGFBP-3 in the serum were associated with improved OS in patients treated with chemotherapy [68]. In colorectal cancer, IGFBP-3 levels were noted to be higher when the disease was stable, and they decreased in cancer progression [69]. These findings highlight the need for further research on IGFBP-3 as a biomarker in the treatment of oncological patients. Nevertheless, in our study, we did not find any significant differences between the levels of IGFBP-3 in the group of patients compared to the control group. Similarly, we did not observe any correlations between levels of IGFBP-3 and the location of the primary lesion, histological grade, clinical stage, Ki-67, and metastasis.

The impact of neoplastic disease on the skeletal system has been extensively described in the literature. It is believed to be destructive not only due to the disease itself but also the treatment applied [70]. To attempt to determine the significance of this process, concentrations of markers of bone turnover were assessed in various malignant tumors. Kowalska et al. suggest that evaluating specific markers and parameters of bone turnover may be useful in the evaluation of the skeletal system in these patients [70]. The researchers observed abnormalities in the selected markers of bone turnover in a group of patients without bone metastases. The effect of the applied treatment on the skeletal system in patients without bone metastases is an interesting aspect, often overlooked in oncological practice [70]. Biochemical markers of bone turnover are also useful in the diagnosis and treatment monitoring of breast cancer with bone metastases [71]. In a study assessing osteocalcin levels in serum and CTx levels in serum and urine before and after treatment with clodronate in patients with metastatic bone disease, significant differences in CTx levels were found between patients with and without bone metastases. Interestingly, two patients who experienced an increase in CTx during treatment showed disease progression in later scintigraphic examinations [71]. According to Lang et al. who assessed the concentrations of selected markers of bone turnover in lung cancer patients with bone metastases, these markers may be useful both for the diagnosis and subsequent treatment selection [66]. Although bone metastasis in neuroendocrine tumors is not common, it can occur [72]. A meta-analysis of 149 studies revealed an 18.4% incidence of bone metastases in NENs. Osteoclastic bone metastases were the most prevalent type, occurring metachronically (61.2%), with the axial skeleton being the primary site of metastasis [73]. In our study of 60 patients with neuroendocrine tumors, 5 patients had bone metastases. However, there were no significant differences in the levels of osteocalcin, osteoprotegerin, and IGFBP-3 between the patients with and without bone metastases. Pheochromocytoma, sympathetic paragangliomas, and high-grade neuroendocrine carcinoma (NEC) have the highest risk of developing bone metastases [74]. According Beom-Jun Kim et al., patients with pheochromocytoma had significantly lower bone mass at the lumbar spine and higher serum levels of C-terminal telopeptide of type I collagen compared with the control group [75]. Milone et al. observed an increased RANKL/OPG ratio in NETs with bone metastases. However, no significant difference in the levels of RANKL was found between the NET patients with and without bone metastases. The researchers suggested that the imbalance in the RANK/RANKL/OPG pathway might be useful in the predicting early bone metastases in patients with NETs [76]. Further studies are needed to investigate this aspect in neuroendocrine tumors.

## 5. Conclusions

Markers of bone turnover, which are protein fragments released into the bloodstream as a result of continuous bone formation and bone resorption processes, are useful markers for the assessment of bone metabolism in cancer. However, due to numerous limitations and their dependence on various factors, they cannot be used in the routine diagnostics of neuroendocrine tumors. Nonetheless, these markers may still contribute to evaluating the skeletal system in patients with NETs. Further research is necessary to determine the potential utility of osteocalcin (OST) and osteoprotegerin (OPG) as biomarkers for neuroendocrine tumors.

### 5.1. Advantages of the Study

According to the available literature (PubMed Database), this is one of the few studies evaluating bone metabolism in NET patients.

### 5.2. Limitations of the Study

This study has potential limitations. First, it is limited by the small number of patients. Second, the age of the study group compared with the control group was significantly greater. Another limitation of this study was the lack of assessment of the concentration of parathyroid hormone or assessment for vitamin D3. However, we plan to monitor the health status of our study groups to deepen our knowledge on the subject. In the future, we plan to extend our research to a larger group of patients and include the parameters of the concentration of parathyroid hormone and assessment for vitamin D3.

## Figures and Tables

**Table 1:** Characteristic of the study group.

			*n* (% of Patients)
Location of the primary tumor	BP-NET cases 17 (28.33%)	Lung	17 (28.33%)
	GEP-NET cases 43 (71.67%)	Pancreas	10 (16.67%)
		Small intestine	5 (8.33%)
		Stomach	5 (8.33%)
		Appendix	4 (6.67%)
		Large intestine	4 (6.67%)
		Rectum	4 (6.67%)
		Duodenum	1 (1.67%)
		Unknown	10 (16.67%)
Histological grade for GEP-NET cases	NET G1		31 (51.67%)
	NET G2		7 (11.67%)
	NET G3		5 (8.33%)
Histological grade for BP-NET cases	Typical carcinoid (TC)		12 (20%)
	Large cell neuroendocrine carcinoma (LC-NEC)		5 (8.33%)
Clinical stage	I		28 (46.67%)
	II		2 (3.33%)
	III		1 (1.67%)
	IV		29 (48.33%)
Ki-67	<3%		18 (30%)
	3% to 20%		6 (10%)
	>21%		5 (8.3%)
	Undetermined		31 (51.7%)
Metastasis	Total		30 (50%)
	Liver metastases		23 (38.3%)
	Lymph node metastases		9 (15%)
	Bone metastases		5 (8.3%)
	Other metastases		9 (15%)

**Table 2:** Demographic and biochemical characteristic of study and control groups with the *p*-values of the Kruskal–Wallis test.

	Study Group	Control Group	
	Mean ± SD	Mean ± SD	*p*
Age [years]	58.02 ± 12.9	35.97 ± 11.5	<0.01
BMI [kg/m^2^]	25.34 ± 4.96	29.29 ± 11.47	0.27
Chromogranin A [µg/L]	414.87 ± 1855.29	-	-
Serotonin [ng/mL]	227.95 ± 280.29	-	-
5-hydroxyindole acetic acid (5-HIAA) [mg/24 h]	12.82 ± 20.57	-	-
Calcium [mmol/L]	2.36 ± 0.18	-	-
Phosphate [mmol/L]	1.12 ± 0.18	-	-
Cortisol [µg/dL]	17.37 ± 6.37	-	-
Glucose [mg/dL]	96.77 ± 33.81	88.18 ± 17.97	0.20
Total cholesterol [mmol/L]	4.98 ± 1.18	4.77 ± 1.49	0.18
Triglycerides [mmol/L]	1.38 ± 0.69	2 ± 2.02	0.45

**Table 3:** Concentration of analyzed parameters of bone turnover in the study and control group.

	Study Group	Control Group	*p*
Osteocalcin [ng/mL]	12.55 ± 11.01	7.50 ± 5.32	<0.01
Osteoprotegerin [pmol/L]	5.70 ± 2.56	4.46 ± 1.34	<0.01
IGFBP-3 [ng/mL]	2885.63 ± 952.33	2877.12 ± 936.40	0.97

## Data Availability

The data used to support the findings of this research are available upon request from the corresponding author, J.S.: janusz.strzelczyk@sum.edu.pl.

## Supplementary Materials

The following supporting information can be downloaded at: https://www.mdpi.com/article/10.3390/jcm12144608/s1, Supplementary Table S1a. Spearman correlation coefficients (r_S_) and probability value (*p*) for the parameters studied in the study group; Supplementary Table S1b. Spearman correlation coefficients (r_S_) and probability value (*p*) for the parameters studied in the control group.
