# Supplementary material for: Selected Parameters of Bone Turnover in Neuroendocrine Tumors—A Potential Clinical Use?
Source: J Clin Med. 2023 Jul 11;12(14):4608. doi: 10.3390/jcm12144608 (PMC10380215; doi:10.3390/jcm12144608)
Supplement: Supplementary file 1 [file jcm-12-04608-s001.zip › jcm-2487221-supplementary.pdf]

Supplementary Table S1a. Spearman correlation coefficients ($r_S$) and probability value (p) for the parameters studied in the study group.

| **Study group** | Age | | BMI | | Chromogranin A | | Serotonin | | 5-HIAA | | Glucose | | Total cholesterol | | Triglycerides | | Osteocalcin | | Osteoprotegerin | | IGFBP-3 | | Histological Grading | | Clinical stage (I-IV) | | Ki-67 | |
|---|---|---|---|---|---|---|---|---|---|---|---|---|---|---|---|---|---|---|---|---|---|---|---|---|---|---|---|---|
| | $r_S$ | p | $r_S$ | p | $r_S$ | p | $r_S$ | p | $r_S$ | p | $r_S$ | p | $r_S$ | p | $r_S$ | p | $r_S$ | p | $r_S$ | p | $r_S$ | p | $r_S$ | p | $r_S$ | p | $r_S$ | p |
| Age | 1.00 | 0.000 | 0.20 | 0.118 | 0.08 | 0.527 | -0.15 | 0.270 | -0.01 | 0.921 | 0.15 | 0.251 | 0.11 | 0.421 | -0.03 | 0.807 | 0.10 | 0.458 | 0.48 | 0.000 | -0.35 | 0.031 | 0.06 | 0.703 | 0.26 | 0.044 | -0.10 | 0.455 |
| BMI | 0.20 | 0.118 | 1.00 | 0.000 | -0.23 | 0.081 | -0.08 | 0.568 | 0.13 | 0.318 | 0.18 | 0.181 | 0.04 | 0.786 | 0.25 | 0.058 | -0.25 | 0.051 | -0.14 | 0.302 | 0.22 | 0.177 | -0.35 | 0.017 | -0.12 | 0.372 | -0.47 | 0.000 |
| Chromogranin A | 0.08 | 0.527 | -0.23 | 0.081 | 1.00 | 0.000 | 0.32 | 0.015 | 0.35 | 0.008 | 0.12 | 0.367 | -0.36 | 0.006 | -0.04 | 0.778 | 0.06 | 0.649 | 0.42 | 0.001 | 0.17 | 0.293 | 0.22 | 0.143 | 0.43 | 0.001 | 0.10 | 0.467 |
| Serotonin | -0.15 | 0.270 | -0.08 | 0.568 | 0.32 | 0.015 | 1.00 | 0.000 | 0.31 | 0.018 | 0.01 | 0.952 | -0.25 | 0.061 | 0.05 | 0.692 | 0.20 | 0.141 | -0.04 | 0.774 | -0.05 | 0.770 | 0.10 | 0.511 | 0.19 | 0.157 | 0.00 | 0.980 |
| 5-HIAA | -0.01 | 0.921 | 0.13 | 0.318 | 0.35 | 0.008 | 0.31 | 0.018 | 1.00 | 0.000 | 0.13 | 0.341 | -0.35 | 0.008 | -0.19 | 0.147 | 0.00 | 0.988 | -0.06 | 0.678 | 0.16 | 0.338 | -0.05 | 0.747 | 0.28 | 0.029 | -0.07 | 0.599 |
| Glucose | 0.15 | 0.251 | 0.18 | 0.181 | 0.12 | 0.367 | 0.01 | 0.952 | 0.13 | 0.341 | 1.00 | 0.000 | -0.04 | 0.748 | 0.20 | 0.131 | 0.02 | 0.870 | 0.25 | 0.052 | 0.10 | 0.542 | 0.37 | 0.010 | 0.16 | 0.236 | 0.19 | 0.156 |
| Total cholesterol | 0.11 | 0.421 | 0.04 | 0.786 | -0.36 | 0.006 | -0.25 | 0.061 | -0.35 | 0.008 | -0.04 | 0.748 | 1.00 | 0.000 | 0.25 | 0.060 | -0.14 | 0.312 | -0.16 | 0.218 | -0.14 | 0.401 | -0.09 | 0.548 | -0.17 | 0.189 | -0.10 | 0.436 |
| Triglycerides | -0.03 | 0.807 | 0.25 | 0.058 | -0.04 | 0.778 | 0.05 | 0.692 | -0.19 | 0.147 | 0.20 | 0.131 | 0.25 | 0.060 | 1.00 | 0.000 | -0.08 | 0.559 | 0.07 | 0.586 | 0.41 | 0.012 | 0.18 | 0.234 | 0.00 | 0.986 | 0.05 | 0.706 |
| Osteocalcin | 0.10 | 0.458 | -0.25 | 0.051 | 0.06 | 0.649 | 0.20 | 0.141 | 0.00 | 0.988 | 0.02 | 0.870 | -0.14 | 0.312 | -0.08 | 0.559 | 1.00 | 0.000 | -0.05 | 0.714 | -0.25 | 0.132 | 0.09 | 0.559 | 0.04 | 0.745 | 0.20 | 0.133 |
| Osteoprotegerin | 0.48 | 0.000 | -0.14 | 0.302 | 0.42 | 0.001 | -0.04 | 0.774 | -0.06 | 0.678 | 0.25 | 0.052 | -0.16 | 0.218 | 0.07 | 0.586 | -0.05 | 0.714 | 1.00 | 0.000 | -0.17 | 0.306 | 0.28 | 0.054 | 0.47 | 0.000 | 0.07 | 0.587 |
| IGFBP-3 | -0.35 | 0.031 | 0.22 | 0.177 | 0.17 | 0.293 | -0.05 | 0.770 | 0.16 | 0.338 | 0.10 | 0.542 | -0.14 | 0.401 | 0.41 | 0.012 | -0.25 | 0.132 | -0.17 | 0.306 | 1.00 | 0.000 | 0.12 | 0.516 | 0.02 | 0.889 | 0.04 | 0.831 |
| Histological Grading | 0.06 | 0.703 | -0.35 | 0.017 | 0.22 | 0.143 | 0.10 | 0.511 | -0.05 | 0.747 | 0.37 | 0.010 | -0.09 | 0.548 | 0.18 | 0.234 | 0.09 | 0.559 | 0.28 | 0.054 | 0.12 | 0.516 | 1.00 | 0.000 | 0.36 | 0.014 | 0.77 | 0.000 |
| Clinical stage (I-IV) | 0.26 | 0.044 | -0.12 | 0.372 | 0.43 | 0.001 | 0.19 | 0.157 | 0.28 | 0.029 | 0.16 | 0.236 | -0.17 | 0.189 | 0.00 | 0.986 | 0.04 | 0.745 | 0.47 | 0.000 | 0.02 | 0.889 | 0.36 | 0.014 | 1.00 | 0.000 | 0.09 | 0.495 |
| Ki-67 | -0.10 | 0.455 | -0.47 | 0.000 | 0.10 | 0.467 | 0.00 | 0.980 | -0.07 | 0.599 | 0.19 | 0.156 | -0.10 | 0.436 | 0.05 | 0.706 | 0.20 | 0.133 | 0.07 | 0.587 | 0.04 | 0.831 | 0.77 | 0.000 | 0.09 | 0.495 | 1.00 | 0.000 |
| Calcium | -0.11 | 0.399 | -0.22 | 0.088 | -0.27 | 0.038 | -0.08 | 0.554 | -0.27 | 0.038 | 0.02 | 0.860 | 0.26 | 0.045 | -0.02 | 0.877 | 0.28 | 0.031 | -0.18 | 0.177 | -0.18 | 0.274 | 0.03 | 0.823 | -0.28 | 0.028 | 0.15 | 0.246 |
| Phosphate | -0.14 | 0.272 | -0.22 | 0.088 | 0.03 | 0.829 | 0.02 | 0.910 | -0.27 | 0.041 | 0.00 | 0.989 | 0.11 | 0.432 | 0.23 | 0.084 | -0.15 | 0.261 | 0.07 | 0.617 | 0.10 | 0.551 | 0.28 | 0.053 | 0.03 | 0.793 | 0.23 | 0.080 |
| Cortisol | 0.09 | 0.497 | 0.03 | 0.849 | 0.18 | 0.174 | -0.02 | 0.899 | 0.08 | 0.544 | 0.34 | 0.008 | 0.09 | 0.508 | 0.08 | 0.546 | 0.03 | 0.797 | 0.14 | 0.278 | 0.02 | 0.881 | -0.01 | 0.953 | 0.00 | 0.974 | -0.06 | 0.627 |

5-HIAA - 5-hydroxyindole acetic acid.

Supplementary Table S1b. Spearman correlation coefficients ($r_S$) and probability value (p) for the parameters studied in the control group.

| **Control group** | Age | | BMI | | Glucose | | Total cholesterol | | Triglycerides | | Osteocalcin | | Osteoprotegerin | | IGFBP-3 | |
|---|---|---|---|---|---|---|---|---|---|---|---|---|---|---|---|---|
| | $r_S$ | p | $r_S$ | p | $r_S$ | p | $r_S$ | p | $r_S$ | p | $r_S$ | p | $r_S$ | p | $r_S$ | p |
| Age | 1.00 | 0.000 | 0.20 | 0.282 | 0.77 | 0.000 | 0.08 | 0.661 | 0.58 | 0.001 | -0.25 | 0.115 | 0.26 | 0.124 | -0.18 | 0.156 |
| BMI | 0.20 | 0.282 | 1.00 | 0.000 | 0.16 | 0.395 | 0.37 | 0.044 | 0.47 | 0.008 | 0.56 | 0.089 | -0.36 | 0.308 | -0.41 | 0.020 |
| Glucose | 0.77 | 0.000 | 0.16 | 0.395 | 1.00 | 0.000 | -0.16 | 0.390 | 0.46 | 0.010 | -0.60 | 0.067 | 0.70 | 0.024 | -0.14 | 0.440 |
| Total cholesterol | 0.08 | 0.661 | 0.37 | 0.044 | -0.16 | 0.390 | 1.00 | 0.000 | 0.36 | 0.051 | -0.50 | 0.141 | 0.00 | 1.000 | -0.24 | 0.206 |
| Triglycerides | 0.58 | 0.001 | 0.47 | 0.008 | 0.46 | 0.010 | 0.36 | 0.051 | 1.00 | 0.000 | -0.30 | 0.400 | 0.60 | 0.067 | -0.10 | 0.599 |
| Osteocalcin | -0.25 | 0.115 | 0.56 | 0.089 | -0.60 | 0.067 | -0.50 | 0.141 | -0.30 | 0.400 | 1.00 | 0.000 | 0.04 | 0.839 | 0.48 | 0.002 |
| Osteoprotegerin | 0.26 | 0.124 | -0.36 | 0.308 | 0.70 | 0.024 | 0.00 | 1.000 | 0.60 | 0.067 | 0.04 | 0.839 | 1.00 | 0.000 | -0.05 | 0.751 |
| IGFBP-3 | -0.18 | 0.156 | -0.41 | 0.020 | -0.14 | 0.440 | -0.24 | 0.206 | -0.10 | 0.599 | 0.48 | 0.002 | -0.05 | 0.751 | 1.00 | 0.000 |
